# Ecological niche partitioning in a fragmented landscape between two highly specialized avian flush-pursuit foragers in the Andean zone of sympatry

**DOI:** 10.1038/s41598-020-78804-2

**Published:** 2020-12-16

**Authors:** Piotr G. Jablonski, Marta Borowiec, Jacek J. Nowakowski, Tadeusz Stawarczyk

**Affiliations:** 1 Laboratory of Behavioral Ecology and Evolution, School of Biological Sciences, Seoul National University, 08-826, Seoul, South Korea; 2 Museum and Institute of Zoology, Polish Academy of Sciences, Wilcza 64, 00-679 Warsaw, Poland; 3 Museum of Natural History, University of Wroclaw, Sienkiewicza 21, 50-335 Wroclaw, Poland; 4 Department of Ecology and Environmental Protection, University of Warmia and Mazury in Olsztyn, Plac Łodzki 3, Olsztyn, Poland

**Keywords:** Behavioural ecology, Biogeography, Conservation biology, Evolutionary ecology, Tropical ecology, Speciation

## Abstract

In the Andes, pairs of ecologically similar species are often separated by narrow elevational sympatry zones but the mechanisms mediating sympatry are not fully understood. Here, we describe niche partitioning within a sympatry zone in a fragmented Andean landscape between two closely related flush-pursue species: a high-elevation montane forest dweller, (*Myioborus melanocephalus*), and a mid-elevation montane forest dweller, (*M. miniatus*). As all flush-pursuers use very similar hunting techniques involving visual displays to flush and pursue insects in air, and benefit from being the “rare predators”, ecological sorting between species in sympatry zones should allow their co-existence. We found that both species occupied vegetation resembling their typical allopatric habitats: a mosaic of pastures, clearings, and shrubs with small proportion of high trees for *M. melanocephalus*, and dense high forests with high proportion of trees, lower irradiance and higher humidity for *M. miniatus*. *M. melanocephalus* often foraged in bushes and at lower heights, whereas *M. miniatus* often foraged in tree crowns. The two species differed relatively little in their foraging technique. These results demonstrate how ecological sorting permits species of divergent elevational distributions and habitats to successfully coexist in sympatric zones where habitat diversity allows both species to find their preferred habitat.

## Introduction

In mountainous areas of the tropics many species are distributed in allopatric elevational zones with occasional narrow zones of sympatry between species^1,2^. Researchers have studied this phenomenon for years, focusing on the reasons for such an elevational segregation of species^1,3–5^. There are three non-exclusive and likely interacting mechanisms that may contribute to the origin and maintenance of elevational divergence^6^: (1) stochastic processes occurring on evolutionary time scale and resulting in higher degree of elevational divergence among the evolutionary older species, (2) elevational divergence actively driven by direct competitive interactions in zones of sympatry and (3) ecological sorting mechanisms that permits species of sufficiently divergent elevational distributions and ecological niches in allopatry to successfully attain sympatry following secondary contact in a zone of sympatry^7^. Observations in the zone of sympatry are crucial in evaluation of these hypotheses, especially the “competition” and “ecological sorting” mechanisms. Interspecific aggression in sympatry zone within pairs of closely related species of specialized and relatively similar foraging ecology may suggest that competition mechanisms are involved. However, if the two species that use similar foraging technique do not interact aggressively while foraging in different habitats of the local environment in their region of elevational sympatry then ecological sorting mechanism is likely involved. Here, we use observational data in a zone of sympatry between two highly specialized foragers from genus *Myioborus,* the Spectacled Whitestart (*M. melanocephalus*)^8^ and the Slated-throated Whitestart (*M. miniatus*)^9^, to focus on several aspects of the ecological sorting between the two species.

The two species belong to a special class of insectivorous birds—the flush-pursuers. Flush-pursuers use a foraging strategy during which a prey is visually startled, flushed and pursued by a bird^10^. All members of the genus *Myioborus* have black-and-white tail pattern which is displayed during hopping on branches with erected and fanned tail (for video see Refs.^11,12^). Adaptive significance of plumage pattern of *Myioborus* is relatively well known^13–19^. This specialized foraging behavior could have evolved through the “rare-enemy” effect^13,20^ as an adaptation of a relatively rare predator to exploit prey anti-predatory escape reactions shaped by selection for avoidance of more common predators. It was proposed that flush-pursuing birds exploit the sensitivity of their prey’s giant escape neurons to looming contrasting stimuli^21–23^, which are typically associated with approaching insectivores from whom the prey escape by jumping or flying away. By foraging with outspread wings and tail, and by presenting spots of contrasting plumage the flush-pursue birds are able to “overstimulate” and to trigger escapes in their prey, which then is pursued and captured in the air. Although the warblers of the genus *Myioborus* and their prey became a model system to study the flush-pursue foraging, the flush-pursue strategy is also observed among other tropical and subtropical birds on every continent. For example, the Austral-Asian genus *Rhipidura* also includes pairs of upper montane (e.g. *Rhipidura atra*;^24^) and lower montane (e.g. *Rhipidura brachyrhyncha*;^25^) flush-pursuer species with elevational distributions^6^ resembling that of *Myioborus* in the Andes. Similar situation appears to exist between the lower elevation flush-pursuer *Trochocercus nitens*^26^ and the higher elevation species, *Elminia longicauda*^27^ in Africa^28^. Hence, by studying genus *Myioborus*, we hope to propose hypotheses relevant to the processes occurring among all tropical and subtropical flush-pursuers with different elevational distributions and sympatric overlap zones of their elevational ranges.

**Figure 1:**
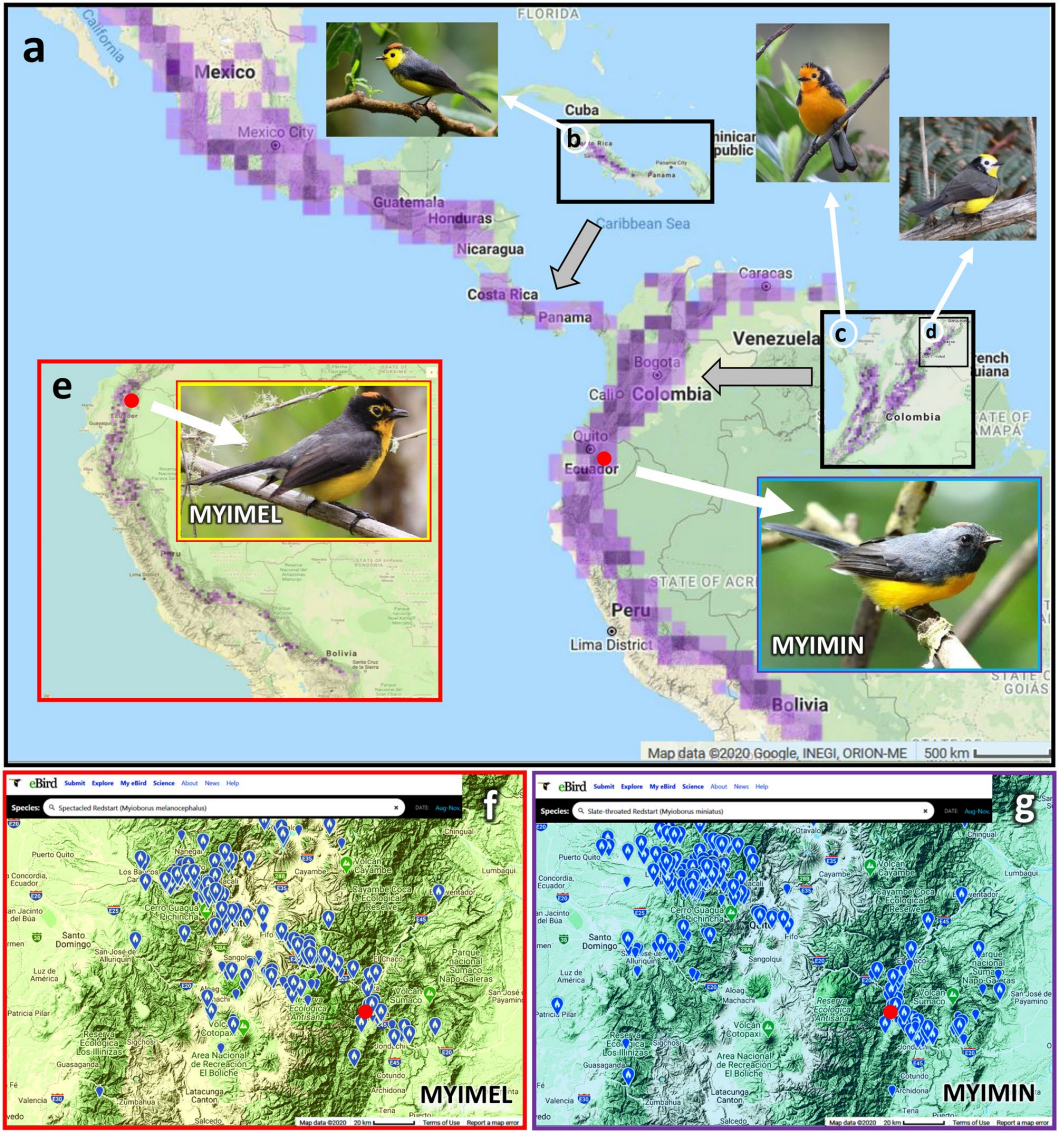
Distribution of some *Myioborus* flush-pursuers in the Andes. (**a**) Wide geographic range of the low-elevation species, *M. miniatus* (MYIMIN); (**b**) high elevation species from Central America, *M. torquatus* and its geographic range; (**c**) high elevation species from Colombia, *M. ornatus* and its geographic range; (**d**) high elevation species from Colombia, *M. albifrons* and its geographic range; (**e**) high elevation species from Ecuador, Peru and Bolivia, *M. melanocephalus* (MYIMEL) and its geographic range; (**f**) distribution of observation records of the high-elevation species, *M. melanocephalus*, in the geographical region of Ecuador where the study site (red circle) is located during August—November season, which is approximately equivalent to the breeding season; (**g**) distribution of observation records of the high-elevation species, *M. melanocephalus*, in the geographical region of Ecuador where the study site (red circle) is located during August—November season, which is approximately equivalent to the breeding season. All maps come from screenshots from eBird^46,47^: images provided by eBird^47^ (www.ebird.org) and created on 24.07.2020. Photo in (**e**) by Jacek Nowakowski. The remaining photos of birds are covered by Creative Commons licenses. Photo in (**a**; MYIMIN) and photo in (**b**)—were taken by “Cephas” and are covered by the Creative Commons Attribution-Share Alike 4.0 International license (https://creativecommons.org/licenses/by-sa/4.0/), and the files are located at: https://commons.wikimedia.org/wiki/File:Myioborus_miniatus_Monteverde_01.jpg; https://commons.wikimedia.org/wiki/File:Myioborus_torquatus_Santa_Elena.JPG. Photo in (**c**) taken by Felix Uribe (Colombia) is covered by Creative Commons Attribution 2.0 Generic license (https://creativecommons.org/licenses/by/2.0/deed.en), and is located at https://commons.wikimedia.org/wiki/File:Myioborus_ornatus_-_Abanico_cariblanco_-_Golden-fronted_Whitestart_(8872557662).jpg. Photo in (**d**) taken by Ross Tsai is covered by Attribution-NonCommercial-NoDerivs 2.0 Generic (CC BY-NC-ND 2.0) License (Link to license: https://creativecommons.org/licenses/by-nc-nd/2.0/), and is located at https://www.flickr.com/photos/rosstsai/5649595588/in/photolist-KWUCyT-qj1qFa-XkR5yi-9ecsBx-GGq93-ew3bh7-i6Eoh8-8SLep4-8SPigG-nQTwjL-eitbvA-xf292n-bDZb93-sCwGJs-hU4BWZ-byxxZb-dPbvbQ-bsGXkR-L9bxNL-4DgNT6-kDBfnX-8TCCWw-8TzwdD-diX3fH-X8YKW4-whZNS1-diYUBx-bw1tGz-8SLeGK-diMtfL-5hqsVu-5T9W87-RSzHc7-HUaPU1-9rLYoB-nhmDjw-nmaynT-64LMHG-W1gKxb-DYZeRW-DL9Ubm-9rRnHj-6cAWCf-23db3Rb-df9VcG-59eiAm-2fm2SRJ-iTR1zU-cCGYLS-9BeDWu.

Genus *Myioborus* comprise 12 insectivorous species living in humid montane forests throughout the American tropics and subtropics^29–31^. As all *Myioborus* flush-pursuers use very similar hunting techniques and benefit from being the “rare predators” within a local ecological guild of insectivorous birds^13,14^, we expect that any two different species of flush-pursuers would benefit from spatial separation into different local habitats in sympatry zones because this could benefit their foraging. Sympatry zones between different *Myioborus* species occur at middle elevations in the Andes (Fig. 1), and are the outcome of evolutionary and ecological processes. Genus *Myioborus* colonized the Neotropical montane habitats in Central and South America by a rapid southward movement through Panamanian land bridge, and subsequently the local populations became isolated, especially at higher elevations^30^. The isolation of *Myioborus* populations in high mountain regions promoted rapid allopatric speciation events^30^ producing a series of high-elevation species such as *Myioborus torquatus* (Fig. 1b;^32^), *M. ornatus* (Fig. 1b;^33^), *M. albifrons* (Fig. 1b;^34^) and *M. melanocephalus* (Fig. 1e;^8^). The isolation among the lower elevation populations was less pronounced and led to intraspecific differentiation within the low-elevation species, the Slate-throated Whitestart^19^ ranging from North/Central America (South of USA/Mexico) to Bolivia (Fig. 1a;^9^). This situation, with one geographically diverse species at lower elevations and a series of separate species at higher elevations, with occasional sympatry zones between them at middle elevations, is typical for the Andean birds^35^ as exemplified by studies on finches, Emberizidae^7,36^ and *Metallura* hummingbirds^5^.

In allopatry, the Slate-throated Whitestart and the Spectacled Whitestart differ in the type of habitat used owing to different vegetation present at different elevations. In Ecuador, the Slate-throated Whitestart occupies lower altitudes, from 800 to 2300 m above sea level, locally up to 2600 m a. s. l. This overlaps with the occurrence of subtropical montane forest^37–39^ (examples of a habitat from the elevational range of *M. miniatus* in Ecuador can be viewed online^40,41^). The Spectacled Whitestart occurs at higher altitudes, mainly 2300–4000 m above sea level^39^ where habitats are generally more open with lower vegetation^37,38^ (an example of such a vegetation can be viewed online^42^).

In some regions, the Slate-throated Whitestarts co-occurs sympatrically with the local high-elevation *Myioborus* species such as the Golden-fronted Whitestart *M. ornatus* in Colombia^31^, the Collared Whitestart *M. torquatus* in Costa Rica and Panama^43,44^, and the Spectacled Whitestart in Ecuador and Peru^39,45^. Recent data from eBird^46,47^ (Fig. 1f, g) show that the ranges of *M. miniatus* and *M. melanocephalus* overlap on both the western and eastern slopes of the Andes in the area of Northern Ecuador near Quito, including the study site (Fig. 1f,g). At the site of our research (red circle in Fig. 1a, e, f, g), in the Antisana volcano massif, the range of the Spectacled Whitestart extends down to 2053 m and the two species are sympatric^48^. This creates an opportunity to study their ecological niches in sympatry. It has not been determined how two specialized flush-pursuer species that use the “rare enemy” strategy divide local habitats in their zones of elevational sympatry, but such a division is expected due to differences in typical characteristics between habitats of high and low elevation species.

Here, we focus on two aspects of ecology and ethology of the two species, *M. miniatus* and *M. melanocephalus*, in their zone of sympatry: (1) habitat use and (2) foraging behavior. Additionally, we report absence of any observational evidence for direct competitive or aggressive interactions between the two species during foraging. We use these data to determine if and how ecological sorting permits the two species of divergent elevational distributions and habitats to successfully coexist in disturbed landscapes where habitat diversity may allow both species to find their preferred habitat.

## Results

### Characteristics of breeding territories

**Figure 2:**
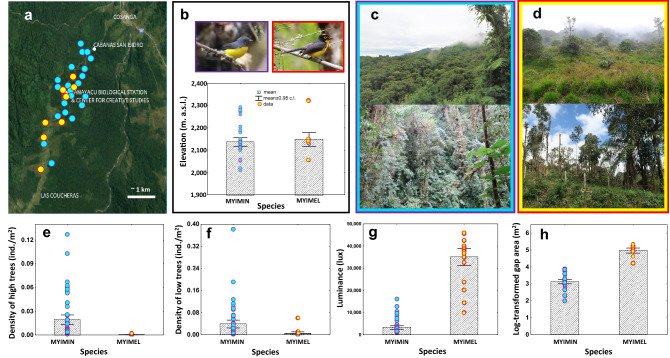
Characteristics of territories of *Myioborus miniatus* (MYIMIN; blue filled symbols with violet edges) and *Myioborus melanocephalus* (MYIMEL; yellow shading, red edges) at the study site. (**a**) spatial distribution of territories shown on the map of the study area; (**b**) distribution of the territories of *M. miniatus* and *M. melanocephalus* with respect to the elevation; (**c**) photos of typical habitats of *M. miniatus* at the study site; (**d**) photos of typical habitats of *M. melanocephalus* at the study site; (**e**) Comparison of density of trees higher than 15 m in territories of *M. miniatus* and *M. melanocephalus*; (**f**) Comparison of density of trees lower than 15 m in territories of *M. miniatus* and *M. melanocephalus*; (**g**) Comparison of luminance in territories of *M. miniatus* and *M. melanocephalus*; (**h**) Comparison of forest gap surface area in territories of *M. miniatus* and *M. melanocephalus*. Based on *n* = 63 and *n* = 24 points measured in the territories of *M. miniatus* and *M. melanocephalus*, respectively. Photos by Jacek Nowakowski. Map Image in (A) retrieved from Google Maps on 16 October, 2019 (Imagery by TerraMetrics, Map data 2019). Summary statistics for variables presented in (**e**–**h**) are presented in the Supplementary Table S1.

**Figure 3:**
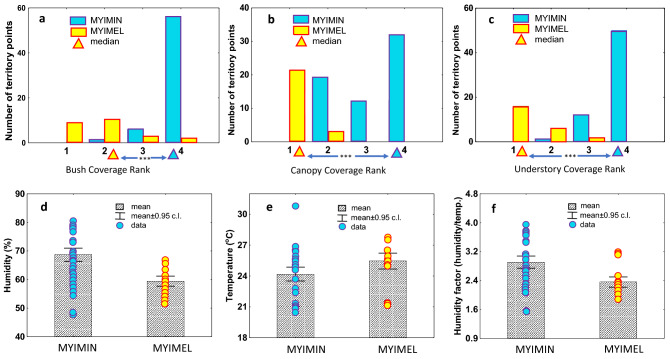
Characteristics of territories of *Myioborus miniatus* (MYIMIN; blue filled, violet edges) and *Myioborus melanocephalus* (MYIMEL; yellow shading, red edges) at the study site. Comparison of distributions of bush coverage rank (**a**), canopy coverage rank (**b**) and understory coverage rank (**c**), and comparison of humidity (**d**), temperature (**e**) and humidity factor (**f**) between the two species. Based on *n* = 63 and *n* = 24 points measured in the territories of *M. miniatus* and *M. melanocephalus*, respectively. Summary statistics for variables in (**a**–**c**) are presented in Supplementary Table S2, and for variables in (**d**–**f**) are presented in Supplementary Table S1.

We found 21 territories of the Slate-throated Whitestart and 8 territories of the Spectacled Whitestart (Fig. 2a). The two species did not differ in the elevation of their territory locations (Mann–Whitney test; U = 0.586, *P* = 0.555; *n*_*myimin*_ = 21, *n*_*myimel*_ = 8; Fig. 2b), but they differed from each other in all the remaining variables considered (Fig. 2c-h, Fig. 3; Supplementary Tables S1 and S2). All 21 territories of the Slate-throated Whitestart were located in the forest, of which 17 (81%) were deep within the forest stands and 4 territories (19%) were situated on the edges of the forest. All 8 territories of the Spectacled Whitestart were located in the semi-open places transformed by human activity. Six territories were located on pastures with some patches of bushes and trees (75%), and two territories were located at the forest edge on the semi-open slopes of the mountains (25%).

We described vegetation and physical characteristics at 63 locations within *M. miniatus* territories and at 24 locations within *M. melanocephalus* territories (3 locations per territory), and we compared the locations between species. The territories of the Slate-throated Whitestart were characterized by high density of trees (including tall trees above 15 m with dense canopies), high density of understory and bushes, high degree of coverage by bushes, canopy and understory, all of which caused the lower luminance and slightly higher humidity of the habitats compared to the Spectacled Whitestart’s territories (Figs. 2e-h and 3, Supplementary Tables S1 and S2). Some small natural gaps in the stand, which resulted from falling trees, occurred within territories of the Slate-throated Whitestart. Looking at the species difference from the Spectacled Whitestart perspective, the habitat characteristics of the territories of this species differed from the territories of the Slate-throated Whitestart by being located in areas with larger gaps in tree stands, trees growing at much lower density, with low degree of canopy coverage (trees taller than 15 m were more scattered, often single), low understory coverage, higher luminance and slightly lower humidity (Fig. 2e-h; Fig. 3, Supplementary Tables S1 and S2). Discriminant analysis revealed that the territories of the two species can be distinguished from each other based on such habitat characteristics of their territories as canopy coverage, understory coverage, bushes coverage, gap area, density of trees, and luminance (Table 1). Classification functions based on the discriminant analysis models allowed for 100% territory occupancy prediction by the species.

**Table 1:** Models of discriminant analysis to separate the territories of the Slate-throated Whitestart and Spectacled Whitestart based on the descriptions of vegetation, luminance, humidity and temperature.

	Function 1	Lambda—Wilks	F removal	*P*
**Model A: Lambda Wilks' test = 0.020; app. F**_**(13/73)**_** = 275.99; *P* < 0.0001**				
Canopy coverage	− 7.948	0.260	67.361	< 0.001
*Interaction* (Canopy coverage x understory coverage)	3.345	0.397	16.864	< 0.001
Bushes coverage	− 0.819	0.660	12.718	< 0.001
Gap area [log10]	− 1.405	0.776	20.497	< 0.001
Density of trees lower than 15 m [log10]	1.030	0.818	15.801	< 0.001
Constant	3.345			
Eigenvalue	49.149			
**Model B: Lambda Wilks' test = 0.032; app. F**_**(12/74)**_** = 184.49; *P* < 0.0001**				
Canopy coverage	− 3.962	0.477	15.111	< 0.001
Understory coverage	0.096	0.710	16.981	< 0.001
Bushes coverage	− 0.389	0.807	8.551	< 0.001
Gap area [log10]	− 1.154	0.825	20.063	< 0.001
Density of trees lower than 15 m [log10]	0.858	0.870	13.604	< 0.001
Luminance [log10]	− 1.520	0.908	7.404	0.008
Constant	10.434			
Eigenvalue	29.917			
**Model C: Lambda Wilks' test = 0.020; app. F**_**(14/72)**_** = 246.08; *P* < 0.0001**				
Canopy coverage	− 7.206	0.355	42.418	< 0.001
Interaction (Canopy coverage ∙ understory coverage)	3.871	0.397	18.068	< 0.001
Gap area [log10]	− 1.322	0.802	17.320	< 0.001
Bushes coverage	− 0.837	0.810	5.491	0.002
Luminance [log10]	− 1.542	0.909	6.985	0.010
Density of trees higher than 15 m [log10]	0.766	0.942	4.320	0.041
Constant	9.376			
Eigenvalue	47.849			

### Characteristic of foraging sites

**Figure 4:**
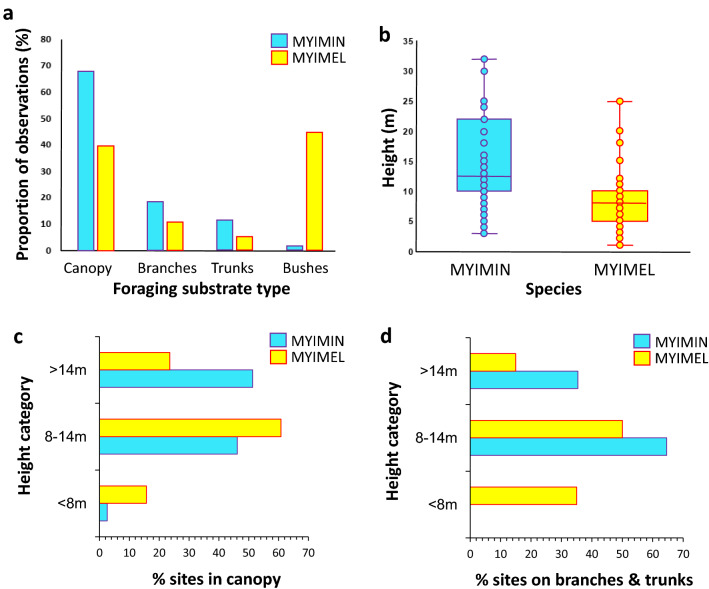
Comparison between *M. miniatus* (MYIMIN) and *M. melanocephalus* (MYIMEL) in their use of foraging sites. (**a**) Distribution of foraging sites (*n* = 168 and *n* = 129 for *M. miniatus* and *M. melanocephalus,* respectively) among the four substrate types; (**b**) Foraging height of *n* = 168 and *n* = 129 foraging sites records of *M. miniatus* and *M. melanocephalus* respectively; (**c**) Distribution of foraging sites in canopies according to the height category for *M. miniatus* (*n* = 117 records) and *M. melanocephalus* (*n* = 51); (**d**) Distribution of foraging sites on branches and trunks according to the height category for *M. miniatus* (*n* = 48 records) and *M. melanocephalus* (*n* = 20). Parts (**c**) and (**d**) are based on values reported in Supplementary Table S3.

The two species differed in the foraging sites used (Fig. 4a; chi-square test: *χ2* = 82.123; *df* = 3; *P* < 0.0001). The Slate-throated Whitestart used mostly foraging sites in the canopy, while the Spectacled Whitestart often foraged in bushes (Fig. 4a). The foraging sequences of the Slate-throated Whitestart’s were recorded at significantly higher locations than the Spectacled Whitestart (Fig. 4b; Mann–Whitney test: *Z* = 9.157, *n*_mymin_ = 168, *n*_mymel_ = 129, *P* < 0.0001), which is clearly associated with higher proportion of foraging by the Slate-throated Whitestart in the tree canopies (Fig. 4a). The difference between species in foraging sites’ height was maintained in the analyses conducted for single foraging records in canopy only (Fig. 4c) or on branches and trunks only (Fig. 4d), but these analyses are also confounded by the lower density of high trees in the territories of *M. melanocephalus* (Fig. 2). The width of the ecological niche based on ten types of foraging site types (3 substrate types × 3 height ranks + bushes; see Materials and Methods and Supplementary Table S3) was slightly larger for the Slate-throated Whitestart (*B* = 3.91) that for the Spectacled Whitestart (*B* = 3.60). Finally, the index of niche overlap (*O*) between the two species (also based on the 10 categories of foraging sites) was *O* = 0.482 (0 means no overlap, and 1 means full overlap).

### Interspecific interactions

During a total of ca 320 h of field work in the three breeding seasons we have not observed any direct indications of contest competition between the two species during foraging. On three occasions, pairs of both species foraged side by side in the same tree and we did not notice any aggressive interactions between them (chasing or fighting).

### Foraging behaviour

**Figure 5:**
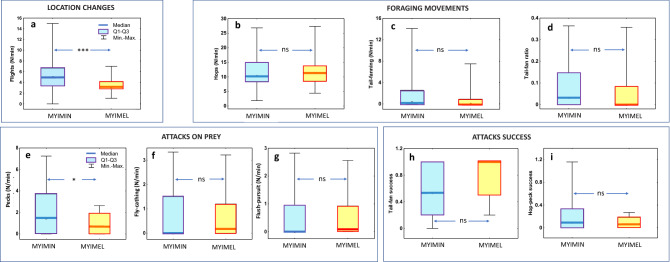
Comparison between *M. miniatus* (MYIMIN) and *M. melanocephalus* (MYIMEL) in their foraging behaviour. (**a**) Foraging location changes described by ***flights*** (nr flights/min); (**b**) Foraging movement rate described by ***hops*** (nr hops/min); (**c**) flush-pursue foraging movement rate described by ***tail-fanning*** (nr of hops with tail fanned/min); (**d**) relative used of the flush-pursue foraging movements as measured by ***tail-fan ratio*** (***tail-fanning***/(***hops*** + ***tail-fanning***); (**e**) attacks on prey by pecking, ***pecks*** (nr/min); (**f**) attacks on prey by ***fly-catching ***(nr/min); (**g**) attacks on prey by flush-pursuing, ***flush-pursuit*** (nr/min); (**h**) flushing success of the fanned tail and wings as measured by ***tail-fan success*** (proportion of tail-fan-hops that resulted in pursuit); (**i**) success in detecting and attacking stationary prey as measured by ***hop-peck success*** (proportion of hops that resulted in pecking). In (**h**), *n* = 20 and *n* = 12 for *M. miniatus* and *M. melanocephalus*, respectively. In all other panels, *n* = 39 and *n* = 26 for *M. miniatus* and *M. melanocephalus*, respectively. ***Differences statistically significant at *P* < 0.001; *Differences statistically significant at *P* < 0.05; ns differences not statistically significant; box charts: line—mean; box—SE; whiskers—95% confidence limits. Full set of statistical analyses is reported in Supplementary Table S4 in Supplementary Materials Part 3.

In general, the Slate-throated Whitestart changed foraging locations more often (Fig. 5a; Mann–Whitney test for variable ***flights***, *Z* = 3.067, *P* = 0.002; Supplementary Table S4) and performed more ***pecks*** per minute (Fig. 5e; Mann–Whitney test *Z* = 1.980, *P* = 0.048; Supplementary Table S4) than the Spectacled Whitestart. The distribution characteristics of the ***fly-catching*** (Fig. 5f) was significantly different between species as revealed by Wald-Wolfowitz runs test (*Z* = 2.919, *P* = 0.004; Supplementary Table S4), indicating that even though the species do not differ in the central tendency (Mann–Whitney test; *Z* = 0.610, *P* = 0.542) they differed in how the ***fly-catching*** was distributed among foraging sequences. The two species did not differ significantly with respect to the remaining foraging variables (Fig. 5, b–d, g–i; full sets of Mann–Whitney tests and Wald–Wolfowitz tests are presented in the Supplementary Table S4).

To determine if these several interspecific differences are also observed when only one foraging substrate is analyzed we looked at the data for tree canopies only. Like for the whole data set, the Slate-throated Whitestart performed significantly more ***flights*** (median: 5.6/min.; Mann–Whitney test *Z* = 2.750, *P* = 0.006), and more ***pecks*** (median: 2.1/min.; Mann–Whitney test *Z* = 2.540, *P* = 0.011) than the Spectacled Whitestart (median values 3.3/min and 0.6/min for flights and pecks respectively) suggesting that perhaps more mobility and higher pecking activity may be the species-specific traits of *M. miniatus*. Additionally, *M. miniatus* had higher ***hop-peck success*** (Mann–Whitney test *Z* = 2.291, *P* = 0.022) than the Spectacled Whitestart. The total attack rate on prey per minute (i.e. ***pecks*** + ***flush-pursuits*** + ***fly-catching***) did not differ between species when all foraging substrates were pooled together (Mann–Whitney test: *Z* = − 1.790, *P* = 0.074; Supplementary Tables S4). However, when only data for canopy were analyzed, the Slate throated Whitestart showed significantly higher ***total attack*** rate (median 3.6/min.) compared to the Spectacled Whitestart (median 1.9/min.; Mann–Whitney test: *Z* = 2.328; *P* = 0.020). The full set comparisons between species in canopy is reported in the Supplementary Table S5. We could not reliably conduct similar tests separately for the remaining three foraging locations (trunk, branch, bush) because of small sample sizes for both or one species.

The results of the GLZ analysis combining multiple variables (as specified in the model; Table 2) revealed that a combination of some aspects of a foraging sequence can be used to predict the species identity of a bird: the more ***flights***, ***pecks***, and ***tail-fanning*** are present in a sequence and the higher in vegetation the foraging sequence happens the more likely the foraging sequence is from the Slate-throated Whitestart.

**Table 2:** Results from the generalized linear model (GLZ) analysis to determine how the probability that the observed foraging sequence belongs to *M. miniatus,* (i.e. when the binary variable *Species* have value 1; MYIMIN = 1, MYIMEL = 0), depends on the *foraging height* and six foraging variables calculated for each foraging sequence (*flights*, *hops*, *tail-fanning*, *pecks*, *flush-pursuit, flycathing*) as well as the type of foraging site.

Variable	Coefficient	SE	Wald statistics	Odds ratio	95% confidence interval for odds ratio	*P*
***Flights ***(nr/min.)	**+ 0.449**	0.1860	5.830	1.567	1.088–2.256	0.016
FORAGING MOVEMENTS						
*Hops* (nr /min.)	− 0.162	0.0870	3.475	0.850	0.717–1.008	0.062
***Tail-fanning*** (nr/min.)	**+ 0.688**	0.3407	4.079	1.990	1.021–3.880	0.043
ATTACKS						
***Pecks*** (nr /min.)	**+ 0.584**	0.2895	4.071	1.793	1.017–3.163	0.044
*Flush-pursuit* (nr/ min.)	− 1.068	0.7109	2.256	0.344	0.085–1.385	0.133
***Height*** of foraging sites (m)	**+ 0.210**	0.0834	6.360	1.234	1.048–1.453	0.012
Intercept	− 2.892	1.4460	3.999	0	0	0.046

The table presents the variables present in the best model (GLZ model, dependk = 6; AIC = 72.192; II likelihood Chi-square = 29.300; *P* = 0.00005). Variables with *P* < 0.05 are marked bold.

To gain some insights (albeit limited due to the data structure and small samples) into the question “Do specific foraging substrates promote different foraging behaviours or foraging performance?”, we compared foraging in canopies to the foraging on the remaining two substrate types for each species separately. The analyses revealed that the Slate-throated Whitestart’s ***peck-success*** (median = 0.2) was significantly (*P* = 0.012) larger, and that birds performed ***pecks*** (median 2.1/min.) at the significantly higher rate (*P* = 0.027) in canopies than in all the remaining substrates used by the species (medians: 0.0 and 0.3/min. for ***peck-success*** and ***pecks***, respectively; more details in Supplementary Table S6). Furthermore, ***tail-fan success*** was also larger (marginally non-significantly at *P* = 0.052) in canopies (median = 0.7) than in the remaining substrates (median = 0.1). Similar comparisons of canopy with the other substrate types (pooled) and canopy with bushes for the Spectacled Whitestart did not reveal statistically significant differences in foraging variables between foraging substrates (more details in Supplementary Table S7).

## Discussion

**Figure 6:**
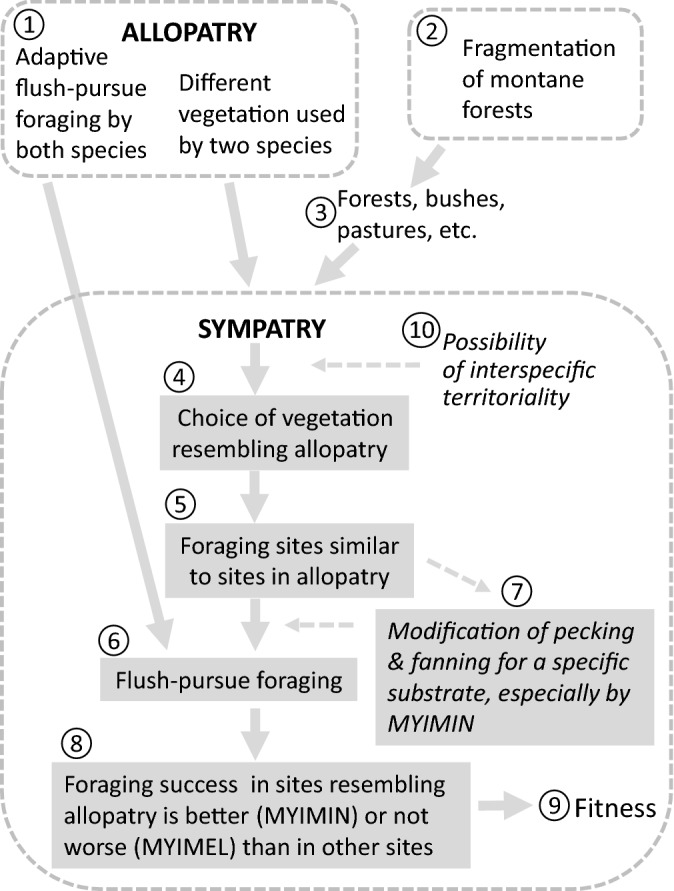
Hypothetical and documented phenomena covered by this research and by literature, and the connections between them to propose a hypothetical mechanism responsible for coexistence of high-elevation and low-elevation *Myioborus* species in the Andean zones of sympatry under anthropogenic changes of landscape. Shaded boxes indicate areas for which we have observational evidence. (1)—in allopatry the two species exhibit similar and likely deeply phylogenetically rooted flush-pursue foraging strategy and they use different species-specific habitats suggesting a possibility of divergent species-specific habitat preferences; (2)—human activities led to fragmentation of the montane forests; (3)—result of fragmentation is a landscape with a mosaic of primary and secondary forests mixed with shrubs and pastures, creating habitat patches resembling allopatric habitats of both species; (4)—species use habitat patches similar to their allopatric habitats and sympatry occurs, which may, hypothetically, be affected by interspecific territoriality (10) based on territorial responses to interspecific songs (observed but not reported here); (5)—each species chooses to forage on substrates and locations that are readily available in territory and following preferences developed during evolution in allopatry; (6)—both species forage in a similar manner, using flush-pursue strategy with small differences consistent with adaptations in allopatry; (7)—species exhibit behavioral plasticity and modify flush-pursue foraging according to foraging substrate (also documented in *M. pictus*^16^); (8)—foraging success is a result of processes in nr 5, 6, 7 and abundance, distribution and profitability of available prey; (9)—the resulting foraging success may affect fitness, which may lead to new evolutionary processes in the anthropogenic zone of sympatry. Notice that this schematic is a hypothesis that should be tested in the future multiple studies at various locations in the Andes for several pairs of low-elevation and high-elevation species (some of which are presented in Fig. 1). See text in Discussion for more explanations.

This is the first quantitative study incorporating detailed foraging information and habitat descriptions in order to evaluate the ecological sorting mechanisms that may contribute to the occurrence of elevational sympatry zones among *M. miniatus* and one of the higher-elevation congeneric flush-pursuers in the Andes (examples in Fig. 1) in situations of anthropogenic habitat fragmentation. We do not have our own or literature-based behavioral data on the mechanisms separating the two studied species in the non-disturbed habitats. However, the distribution-based evidence suggests that competition is involved^4^. Additionally, elevational zones of overlap in mostly undisturbed habitats are relatively narrow: up to 100 m wide between *M. miniatus* and *M. melanocephalus* in Peru^49^, and less than 100 m between *M. miniatus* and *M. torquatus* in Costa Rica^50,51^ compared to at least 300 m wide elevational sympatry zone at our study site (the zone is wider because the 300 m concerns only our study site while the two species seem to occur in sympatry over larger range; Fig. 1f, g). Therefore, we cannot exclude the possibility that different sets of mechanisms separate the Slate-throated Whitestart from its higher-elevation congener at various Andean locations in situations of different degree of anthropogenic disturbance. The hypothetical scenario proposed here assumes anthropogenic fragmentation of the original habitats (Fig. 6) and it can be used as one of the hypotheses considered in the future empirical studies of elevational sympatry zones between low and high elevation flush-pursuers. The results are consistent with the idea that the pre-existing traits of species in allopatry (nr 1 and nr 6 in Fig. 6) combined with the anthropogenic changes in the Andean landscape (nr 2 and 3 in Fig. 6) might have led to sympatry between the two species at our study site located at an elevation that may harbor especially rich and abundant prey fauna as shown for moths and butterflies^52,53^. The territories of the two whitestart species clearly differed in habitat characteristics in the manner generally consistent with the species-specific typical habitats in their zones of allopatry over their entire range: densely forested high-canopy of low elevation montane forests typical for the Slate-throated Whitestart^31^ and more open and fragmented high montane forests typical for the Spectacled Whitestart^31^ (nr 4 in Fig. 6). The presence of denser and taller forest with extended tree canopies in territories of *M. miniatus* at our study site coincided with frequent use of canopies (nr 5 in Fig. 6) for foraging by this species, and with the higher foraging performance in canopies (as reflected in the three variables: pecks, peck-success, and tail-fan success) than in other locations (nr 8 in Fig. 6). However, as we do not have similar quantitative data from allopatry zones adjacent to the study site, we treat our interpretation as the “best-guess” hypothesis considering the available data.

Additionally, between the two species foraging in canopies, *M. miniatus*, for whom canopy is the typical foraging substrate, performed better (as measured by pecks, hop-peck success and total attack) than *M. melanocephalus*, for whom high tree canopies are less typical foraging substrates. The results suggest that in the zone of sympatry the Slate-throated redstart is able to use the habitats and foraging substrates that provide good foraging opportunities. The presence of lower vegetation and more fragmented forests in territories of the Spectacled Whitestart coincided with frequent use of bushes by this species, which did not suffer a decrease in foraging performance in bushes compared to canopies (nr 8 in Fig. 6). We cannot reject the possibility that *M. melanocephalus* might have even experienced better foraging performance in bushes because shrubs and young secondary forests may harbor prey taxa of larger body sizes^54^ and higher abundance^55,56^ than the primary montane forest and its understorey. Hence, our observations are consistent with the hypothesis of ecological sorting, which posits that niche differences that originally evolved in allopatry permit coexistence of the two species in their zone of sympatry where habitat variation created by anthropogenic habitat fragmentation allows for both species to find the preferred habitat characteristics^6,7^. The foraging niche width was wider in the Spectacled Whitestart compared to the Slate-throated Whitestart, which is also consistent with differences in vegetation between the species as a relatively more diverse (less uniform) vegetation structure was detected in the territories of the Spectacled Whitestart than the Slate-throated Whitestart. The ecological niche overlap index based on foraging site location characteristics was in the range of values indicating lower/intermediate degree of niche overlap^57^ confirming that the two species differ in their ecological niches. All these results combined with the lack of any observed aggression between the two species are consistent with the ecological sorting mechanism^6^.

Finally, we cannot exclude the possibility that aggressive interactions among singing territorial males (nr 10 in Fig. 6), demonstrated in the *Rhipidura* flush-pursuers through song-playback experiments^58^, may contribute at the specific time of establishing breeding territories to the local ecological separation of neighboring territories between the two *Myioborus* species or even to establishing borders between the two species’ elevational ranges. The latter should be considered as *M. miniatus* elevational range went up-slope in the mountain range Cerros del Sira, where the high-elevation congener, *M. melanocephalus*, is absent even though the habitat typical for *M. melanocephalus* (not *M. miniatus* !) extends to lower elevations than typically in the Andes^4^. Detailed results from playback experiments on our study species to address this issue will be reported separately (Nowakowski, Borowiec, Stawarczyk, personal communication), but preliminary overview of the data indicates that interspecific responses to experimental playbacks of territorial songs are observed between the two species and may contribute to the local spatial separation of territories into different habitats.

While the two sympatric species clearly differed in the territory properties and foraging site characteristics (on which the ecological niche overlap calculation was based), they differed relatively little in how they behaved during foraging (nr 6 in Fig. 6). Furthermore, the few observed behavioral differences in foraging can be also viewed, at least partly, as a consequence of differences in the types of sites the two species use for foraging, which is a direct consequence of differences in habitats of their territories (nr 7 in Fig. 6). As the Slate-throated Whitestart foraged mainly in tree crowns at higher heights above the ground it performed more flights between separate foraging locations that may be relatively distant from each other (e.g. different trees or distant branches). As the Spectacled Whitestart foraged more often in the lower and thicker bushy vegetation where frequent flyovers may not be necessary it performed less flights between locations.

The Slate-throated Whitestart pecked at prey at the higher rate than the Spectacled Whitestart (nr 8 in Fig. 6). We can exclude the potential effect of search speed differences because the hopping rates were similar between the two species. We hypothesize that this difference may be related to either higher density or better visibility of their prey in the Slate-throated Whitestart’s typical foraging sites (high canopy) than in bushes, or to differences in the detected prey item’s threshold body size/profitability at which the two species decide to attack. It may be reasonable to consider that a species that typically encounters relatively large prey items shall have a higher threshold of profitability and will attack fewer of the small prey. As there are indications that some arthropods’ body size and abundance is larger at the elevations typical for the Spectacled Whitestart^53,59,60^ we hypothesize that the Spectacled Whitestart have a different threshold and might have indeed ignored some of the smaller prey. However, due to paucity of data on the diet composition of both whitestarts in Ecuador and lack of data on abundance and size of typical prey we can only speculate about these issues. The differences (more flights, more pecks by *M. miniatus*) were still observed for comparisons within canopy only, where additionally higher proportion of hops results in pecking (***hop-peck success***) for *M. miniatus* than for *M. melanocephalus*. Therefore, it appears that these behavioral traits distinguish *M. miniatus* from *M. melanocephalus*, and they can possibly be adaptations to typical foraging habitats of *M. miniatus* in allopatry (nr 1 in Fig. 6).

The positive significant coefficients in the GLZ model (Table 2) revealed that high frequency of location changes (***flights)***, high rate of ***pecking*** and high rate of ***tail-fanning*** in a sequence are all indicative of the Slate-throated Whitestart. Hence, this analysis revealed that more frequent ***tail-fanning*** can be also viewed (when combined with more frequent ***flights*** and ***pecking***) as the distinguishing feature of *M. miniatus.* The more frequent use of spread tail for flush-pursue foraging by the Slate-throated Whitestart might have been promoted by more shady habitats with low luminance because contrasting patterns on the tail may be more effective at flushing the prey when presented in darker habitats and/or against darker background^15,22^ (nr 7 in Fig. 6). Additionally, we suspect that the Spectacled Whitestart foraging in the dense bushes may rely to a higher degree on substrate-borne vibrations from a moving bird that can cause the prey to escape^21^ without the need for the visual displays from the fanned tail (nr 7 in Fig. 6).

Similar sympatry zones within pairs of *Myioborus* flush-pursuers (some of the pairs are presented in Fig. 1) may increase in the near future due to anthropogenic changes in the environment (nr 2 and 3 in Fig. 6). Research conducted on the eastern slopes of the Andes in Peru^58^ showed that from 1980 to 2009 the coverage of the cloud forest has decreased over this period from 75 to 60%, and the area occupied by scrub and pasture has increased from around 25–40%. Anthropogenic changes are taking place in Ecuador, but their scale is not precisely determined. As the anthropogenic fragmentation of forested areas create new habitats suitable for the Spectacled Whitestart at lower elevations, this type of anthropogenic habitat changes will likely cause a descent of the Spectacled Whitestart to lower elevations into the elevational zone typical for the Slate-throated Whitestart. Therefore, we predict that new zones of elevational sympatry will originate when the Spectacled Whitestart moves down into those new open habitats. A similar process was already noted in Costa Rica, where the narrow zone of overlap between the low-elevation species, the Slate-throated Whitestart, and the high elevation species, the Collared Whitestart (*M. torquatus*), was modified by anthropogenic habitat changes^43^. These processes are expected to occur throughout the Andes in other pairs of *Myioborus* flush-pursuers with distinct elevational ranges (i.e. involving *M. miniatus* and the corresponding local congener from high elevations), as well as among similar pairs of flush-pursuers in other tropical and subtropical mountains world-wide (e.g. among *Rhipidura* in Austral-Asian region^58^. Hence, based on our research we hypothesize that the ecological and behavioral processes considered here (Fig. 6) will intensify as the birds respond to climate and environmental changes of tropical Andes leading to changes in their elevational ranges^61–63^. However, as long as we do not have more empirical evidence from the other locations where the sympatry zones occur between those two species we can only treat our hypothetical scenario (Fig. 6) as the possibility that should be considered in the future studies at various locations with various types of habitat disturbance. Our results illustrate how the future studies may benefit from collecting and analyzing detailed foraging variables, and we encourage field ornithologists to conduct and publish similar studies at different Andean locations where *M. miniatus* co-occurs with other high-elevation *Myioborus* species, as well as at locations in Austral-Asian region where *Rhipidura* flush-pursuers co-occur in elevational sympatry zones.

## Methods

### Ethics declaration

No animals were captured, harmed or stressed in any way during this study. Observations of birds were conducted from a distance without disturbance in accordance to the laws of Ecuador and in accordance with the rules for researchers at the Yanayacu Biological Station & Center for Creative Studies.

### Study area

The study was carried out in the vicinity of the Yanayacu Biological Station & Center for Creative Studies, 5 km west of Cosanga, prov. Napo, NE Ecuador, at an altitude of ca. 2100 m a.s.l., on the eastern slopes of the Andes (area between Cabanas San Isidro 0°35′24.00S; 77°53′07.08.441W and Las Coucheras 0°37′09.84S; 77°53′58.919W (between − 0.5900, − 77.8853 and − 0.619400, − 77.8997 in Google map coordinates format). The study site is typical of lower montane forests or mid-elevation cloud forest with semi-open spaces such as natural clearings partially covered by *Chusquea* bamboo and pastures with scattered bushes and small trees. The canopy occurs at the height of 20–35 m, understory is well developed and epiphytic growth is abundant and most plants host thick layers of mosses and liverworts. Total annual rainfall ranges from 2300 to 3500 mm, with a drier season lasting roughly from September to February^64^.

### Territory mapping

We searched for territories of the two species along the road from Cosanga to Las Coucheras as well as along three trails going into the forest from the main road (Fig. 2a). Total length of transect was ca. 5.7 km. Territories’ locations along the transect were estimated by measuring position of the birds with GPS and placing their movements on the map. To establish boundaries of the territories, birds in every territory were attracted by song playback and followed by the observer until birds stopped reacting to the stimulation. We did not measure the size of the territories. During all observations we paid attention if any behavioral indications of interspecific aggression occur.

### Habitat description in breeding territories

In each territory of the two species, three points were randomly selected for measurements of environmental features. From every point a distances to nearest trees exceeding height of 15 m and distances to nearest trees with a height of less than 15 m in each of four 90° sectors (determined in accordance with rose directions established with a compass) were measured. Distances greater than 10 m were measured using EDM Leica Rangemaster 1600. Distances from random points to the nearest tree in 90° sectors (quadrant) were used to assess the density of trees in the bird territories according to Point-Quarter Method^65^.

$$\widehat{N}_{p} = \frac{{4\left( {4n - 1} \right)}}{{\pi \sum \left( {r_{ij}^{2} } \right)}},$$

where *N*_*p*_—population density, n—number of random points, π—3.14159, r_ij_—distance between random point and the nearest tree in quadrant.

From each point, the degree of bush coverage, degree of understory coverage and degree of canopy coverage were visually assessed by an observer according to a four level ordinal scale: rank 1 (up to 25% coverage), rank 2 (26–50% ), rank 3 (51–75%), rank 4 (above 75%). In addition, we measured a size of gap area in the stand (area where sky was visible between trees, in m^2^), altitude a.s.l. (using Garmin GPSMap 76S), luminance in lux (using Sekonic Iluminometer i-346, five measurements: one vertical and four horizontal 1 m above the ground and then we used average values), as well as temperature and humidity (using a portable Silva Pro Atmospheric Data Centre). All measurements were carried out between 7:00–10:00 a.m.

### Foraging behaviour

Foraging data were collected during three breeding seasons: 18 September through 10 October 2008, 4 through 23 October 2009 and 30 August through 23 September 2010. Habitat measurements were taken between 28 September and 18 October 2012. All periods of study occurred during the breeding season of both species in Ecuador^48^ when birds were active and vocal. Birds were not individually marked, but we observed all birds occupying all breeding territories we found. During foraging observations each individual was followed until it was lost from view for more than 5 min. All foraging movements were recorded on an audio-recorder including flights (moves between branches or trees by using wings), hops (moves without using wings) in normal posture (labeled here as “hops”), hops in the spread-tail display posture (labeled as „tail-fan”), pecks (hops with gleaning a prey from substrate), flycatching (prey attacks in aerial pursuit not preceded by the “tail-fan”) and flush-pursuit behavior (prey attacks in aerial pursuits preceded by the “tail-fan”). We did not measure the distances of hops or flights length but observations did not suggest large differences between the two species.

Because prey items were small we could not identify them or determine if a given prey attack was successful or not. Therefore, prey attacks instead of successful prey capture were used in analyses of foraging behavior. The total duration of foraging sequences was 3920 s for the Slate-throated Whitestart and 4071 s for Spectacled Whitestart. When the bird was temporarily lost from view this brief period was excluded from the total time of observation. The mean duration of the *n* = 39 separate foraging sequences (total time when the bird was visible) was 100 s (range 16–300 s) for the Slate-throated Whitestart and of *n* = 26 foraging sequences was 151 s (range 20–480 s) for the Spectacled Whitestart. Each observed foraging bout was treated as one sample in the statistical analyses. For each observed sequence, nine variables were determined. They can be grouped into four aspects of foraging behavior:

*LOCATION CHANGES* (a variable describing changes between foraging locations):

- ***flights***: nr of flights/minute—flying from branch to branch or from tree to tree without attacking prey;

*FORAGING MOVEMENTS* (three variables describing movements during foraging within a foraging patch/location such as a tree, bush, branch, etc.):

- ***hops***: nr of hops/minute – number of all hops steadily through vegetation or along branches and tree trunks per minute. This includes hops with tail-fans below, hops followed by flush-pursuit, hops follow by peck and in general all hops.
- ***tail-fanning***: number of tail-fan hops/minute—number of hops in the characteristic spread-tail foraging posture per minute;
- ***tail-fan ratio***: proportion (range 0–1) of tail-fans in all foraging movements calculated as: [nr of tail-fans]/([nr tail fans] + [nr of hops]);

*ATTACKS ON PREY* (three variables describing prey attacks during foraging):

- ***pecks***: nr of pecks/minute—number of hops that were followed by pecks (gleaning) on prey directly from vegetation or other foraging substrates per minute;
- ***fly-catching***: number of flycatching flights/minute—number of prey attacks in aerial pursuits that were not preceded by tail-fan;
- ***flush-pursuit***: nr of flush-pursuits/minute—number of prey attacks in aerial pursuit after tail-fan;

*SUCCESS* (two variables describing how “successful” are the foraging movements; success is defined as an event when attack occurred, hence it indicates a success in detecting a prey that is sufficiently profitable to be attacked; this is not the actual success at capturing the prey):

- ***tail-fan success***: proportion (range 0–1) of tail-fans that were followed by flush-pursuit calculated as: [nr of flush pursuits]/[nr tail fans];
- ***hop-peck success***: proportion (range 0–1) of hops that were followed by a peck (gleaning). calculated as: [nr of pecks]/[nr of hops].

Additionally, we created two variables that are a combination of some of the nine variables:

- ***total attacks***—this variable was calculated for each foraging sequence as a sum: ***pecks*** + ***fly-catching*** + ***flush-pursuit;***
- ***total movements***—this variable was calculated for each foraging sequence as a sum: ***hops*** + ***tail-fanning.***

The total number of behavioral events recorded per minute of a foraging sequence bout (***fights, hops, tail-fanning, pecks, fly-catching, flush-pursuint***) was 22.0/minute (range 7.4–50.1) for the Slate-throated Whitestart and 18.3 /minute (range 9.8–40.5) for the Spectacled Whitestart.

### Foraging height and substrate type

#### Foraging sites

To compare the frequency of use of different foraging substrates and to calculate indices of ecological niche width and overlap, we used 168 and 129 independent records (sightings) for *M. miniatus* and *M. melanocephalus* respectively*.* A single record is an event when a bird was spotted foraging. These records were used to calculate indices of niche breadth and niche overlap. First, these records were used to compare frequency of use of the four different ***foraging substrate types***: tree canopy (thin leafy branches and twigs), tree branches, tree trunks and bushes, ignoring the height information. Next, we used the following categorization based on a combination of four ***foraging substrate types*** and three **height categories**: 1. canopy of trees < 8 m height; 2. canopy of trees 8–14 m height, 3. canopy of trees > 14 m height, 4. trunk of trees < 8 m height, 5. trunk of trees 8–14 m height, 6. trunk of trees > 14 m height, 7. branches (thick unleafy branches) of trees < 8 m height, 8. branches of trees 8–14 m height, 9. branches of trees > 14 m height, and 10. bushes (low vegetation in understory and semi-open habitats). The height of a foraging site was noted with accuracy of 1 m. Heights greater than 10 m were measured using EDM Leica Rangemaster 1600, and the lower ones were estimated by eye. We used Levins’s index of niche width^66^, which was calculated from the data on the use of foraging sites.

$$B = {1}/\sum \left( {p_{i} } \right)^{{2}} ,$$

where *p*_*i*_—share of a particular foraging site type in all foraging sites; *i*: from 1 to 10. Hence, the value of the niche width can vary from 1 for the narrow niche to 10 for the wide niche.

Degree of overlap of resource utilization (foraging niches) between the two species was calculated according to Pianka^67^:

$$O_{jk} = O_{kj} = \frac{{\mathop \sum \nolimits_{i}^{n} p_{ij} p_{ik} }}{{\sqrt {\mathop \sum \nolimits_{i}^{n} p_{ij}^{2} } \mathop \sum \nolimits_{i}^{n} p_{ik}^{2} }} ,$$

where overlaps (*O’s*) are symmetrical and *p*_*ij*_ and *p*_*ik*_ are the proportions of the *i*th resource (foraging places type) used by the *j*th and *k*th species. The index can range from 0 (no overlap) to 1 (full overlap), but in practice for similar avian foraging site distribution data the range of recorded values is approximately from ~ 0.2 to ~ 0.4 for low overlap and from ~ 0.6 to ~ 0.8 for high niche overlap^57^.

#### Height of foraging sequences

If during observations resulting in in foraging sequences, a bird was changing the height of foraging we calculated mean foraging height for every foraging sequence (variable ***mean foraging height*** [m]). Hence the sample sizes for the comparison of ***mean foraging height*** between species are *n* = 39 sequences and *n* = 26 sequences for the Slate-throated Whitestart and Spectacled Whitestart respectively.

### Statistical analysis

#### Territory characteristics

The differences in territory characteristics described by continuous variables with normal distribution were tested with parametric tests: a multidimensional—MANOVA Wilks test, one-dimensional—Student's t-test or Cochran-Cox test. Comparisons for ranked variables were evaluated by non-parametric Mann–Whitney test^68^. Finally, the General Discriminate Analysis (GDA; Refs.^69,70^ between the Slate-throated Whitestart and Spectacled Whitestart territories was based on the following continuous variables: density of trees higher than 15 m, density of trees lower than 15 m, luminance, humidity factor (humidity/temperature), the size of gaps in the stand, as well as on the ranking variables: the degree of bushes coverage, degree of understory density and degree of canopy density. In models we also used the humidity factor (humidity/temperature), that has higher values for more humid and cooler locations, and lower values for more dry and hot locations. This index is similar to the Sialininov’s hydrothermal index used in meteorology^71^ and also avian ecology^72^. Data on the density of trees and luminance were introduced into the model as log-transformed variables, for compatibility with distribution of other variables with normal distributions. Stepwise model selection was used^70^.

#### Foraging sites

The general comparison between the species in their use of the four foraging site types (canopy, trunk, branches and bushes) was conducted on *n* = 168 records from the Slate-throated Whitestart and *n* = 129 records from the Spectacled Whitestart using chi-square test, and chi-square with Yates' correction for comparison the distribution of species in one site type^68^. We also used these records to compare (chi-square test) the species with respect to the frequency of use of the three height classes (< 8 m, 8–14 m, > 14 m) within a specific substrate category: canopy and, separately, branches and trunks.

#### Foraging behaviour

We used Mann–Whitney test to compare the two species with respect to the nine foraging variables. As each variable represents a different specific hypothesis we did not to use correction of the significance level due to multiple comparisons (e.g. Bonferroni correction;^73^. Use of these type of corrections have been criticized^74^ and we believe it is not helpful in our case because we are interested in separately testing different specific hypotheses associated with the different analyzed variables. The Mann–Whitney test focuses on comparisons of the central tendency (median) between species. We also used the Wald-Wolfowitz test to further determine if the species differ in the distribution properties of the variable’s recorded values. Finally, we used generalized linear models (with binomial distribution of dependent variable and log-link) to determine if and how the probability that the observed foraging sequence belongs to *M. miniatus,* (i.e. when the binary variable *Species* has value 1; MYIMIN = 1, MYIMEL = 0), depends ***foraging height***, ***flights***, ***hops***, ***tail-fanning***, ***pecks***, ***flush-pursuit, flycatching***, and ***foraging substrate type*** (canopy, trunk and branches, and bushes). Two-way interactions among the foraging variables (***flights, hops, tail-fanning, flush-pursuit, flycatching***), and the interaction term between ***foraging height*** and ***foraging substrate type***, were allowed in the initial models. The Akaike information criterion (AIC)^75^, corrected for small samples, was used to compare the candidate models and to select the best model^76^.

## Supplementary information

Supplementary Information.

## Data Availability

The datasets generated and analyzed during the current study are available upon request requested from JJN.
