# Supplementary material for: Ecological niche partitioning in a fragmented landscape between two highly specialized avian flush-pursuit foragers in the Andean zone of sympatry
Source: Sci Rep. 2020 Dec 16;10:22024. doi: 10.1038/s41598-020-78804-2 (PMC7745022; doi:10.1038/s41598-020-78804-2)
Supplement: Supplementary file 1 — Supplementary Information. [file 41598_2020_78804_MOESM1_ESM.pdf]

SUPPLEMENTARY MATERIALS TO:

Ecological niche partitioning in a fragmented landscape between two highly specialized avian flush-pursuit foragers in the Andean zone of sympatry

Piotr G. Jablonski[1,2], Marta Borowiec[3], Jacek Józef Nowakowski[4] , Tadeusz Stawarczyk[3]

[1] Laboratory of Behavioral Ecology and Evolution, School of Biological Sciences, Seoul National University, 08-826, Seoul, South Korea

[2] Museum and Institute of Zoology, Polish Academy of Sciences, Wilcza 64, 00-679, Warsaw, Poland

[3] Museum of Natural History, University of Wroclaw, Sienkiewicza 21, Wroclaw 50-335, Poland

[4] Department of Ecology and Environmental Protection, University of Warmia and Mazury in Olsztyn, Plac Łodzki 3, Olsztyn, Poland

Corresponding author:
Jacek J. Nowakowski: jacek.nowakowski@uwm.edu.pl

SUPPLEMENTARY MATERIALS PART 1 -*DESCRIPTION OF TERRITORIES*.. 2

SUPPLEMENTARY MATERIALS PART 2 – *ECOLOGICAL NICHE DETAILS*... 3

SUPPLEMENTARY MATERIALS PART 3
– *COMPARISON OF FORAGING VARIABLES BETWEEN SPECIES*.. 4
– *COMPARISON OF FORAGING VARIABLES BETWEEN SUBSTRATES*.. 6

SUPPLEMENTARY MATERIALS PART 1 -*DESCRIPTION OF TERRITORIES*

Supplementary Table S1. Comparison between the Slate-throated Whitestart (MYIMIN) and the Spectacled Whitestart (MYIMEL) of the characteristics of the vegetation and physical properties of their territories. Parametric testing (*t*-test) was used for log-transformed (log_{10}) tree density, luminance, and gap area. Mann-Whitney test was used for humidty, temperature, and humidity factor. The variables are also presented graphically in Fig. 2e-h and Fig. 3d-f in the main text. Based on n=63 and n=24 sampling locations in the territories of *M. miniatus* and *M. melanocephalus*, respectively. *** p <0.001, ** p <0.01.

Variable	Species	Mean	*SD*	Minimum	Maximum
Density of trees higher than 15 m (***)	MYIMIN	**19.17**	*24.37*	1.10	127.32
[nr trees/1000 m^2]	MYIMEL	**0.43**	*0.35*	0.16	1.85
Density of trees lower than 15 m (***)	MYIMIN	**38.55**	*55.99*	0.74	381.97
[nr trees/1000 m^2]	MYIMEL	**4.86**	*12.14*	0.63	60.63
Luminance (***)	MYIMIN	**3,220**	*3,102*	963	16,106
[lux]	MYIMEL	**35,008**	*9,123*	10,060	46,000
Relative Humidity (***)	MYIMIN	**68.67**	*9.19*	47.7	80.5
[%]	MYIMEL	**57.68**	*4.17*	51.2	66.8
Temperature (**)	MYIMIN	**24.18**	*2.69*	20.4	30.8
[ºC]	MYIMEL	**25.45**	*1.84*	21.1	27.7
Humidity factor (**)	MYIMIN	**2.91**	*0.67*	1.55	3.95
[Celsjus/%]	MYIMEL	**2.36**	*0.34*	1.87	3.17
Gap area (***)	MYIMIN	**2,300**	*2,350*	100	7,500
[m^2]	MYIMEL	**118,450**	*65,900*	16,500	226,850

Supplementary Table S2. Comparison of the vegetation cover at sampling locations in the territories of the Slate-throated Whitestart (MYIMIN) and Spectacled Whitestart (MYIMEL) described as ranks (0-4). The ranked data and humidity factor were tested using the Mann-Whitney test; degree of coverage of bushes, canopy and understory according to the scale: 1 – <25%; 2 – <50%; 3 – <75%; 4 – 75–100%. The variables are also presented graphically in Fig. 3a, b, c.; *** p <0.001, ** p <0.01.

Variable	Species	Median	Minimum	Maximum
Bushes coverage (***)	MYIMIN	4	2	4
	MYIMEL	2	1	4
Canopy coverage (***)	MYIMIN	4	2	4
	MYIMEL	1	1	2
Understory coverage (***)	MYIMIN	4	2	4
	MYIMEL	1	1	3

SUPPLEMENTARY MATERIALS PART 2 – *ECOLOGICAL NICHE DETAILS*

Supplementary Table S3. Distribution of foraging records of *M. miniatus* (MYIMIN, n=168) and M. *melanocephalus* (MYIMEL, n=129) among ten foraging site categories. The graphical representation of the data for canopies is in Fig. 4.

Foraging site category	Foraging substrate and height category	Species			
		MYIMIN		MYIMEL	
		N	%	N	%
1	Canopy <8 m	3	**1.8%**	8	6.2%
2	Canopy 8-14 m	54	32.1%	31	24.0%
3	Canopy >14 m	60	35.7%	12	9.3%
4	Branches <8 m	0	0.0%	3	2.3%
5	Branches 8-14 m	14	8.3%	7	5.4%
6	Branches >8 m	14	8.3%	3	2.3%
7	Trunk <8 m	0	0.0%	4	3.1%
8	Trunk 8-14 m	17	10.1%	3	2.3%
9	Trunk >14 m	3	1.8%	0	0.0%
10	Bushes	3	1.8%	58	45.0%

SUPPLEMENTARY MATERIALS PART 3

COMPARISON OF FORAGING VARIABLES BETWEEN SPECIES

Supplementary Table S4 provides all tests for comparisons between species presented in Fig. 5 of the main text, and Supplementary Table S5 provides analogical comparisons for only one type of foraging substrate: tree canopy (CA).

The analyses in Supplementary Tables S6 and S7 focus on comparisons between different foraging substrates within one species: separately for the Slate-throated Whitestart (MYIMIN; Supplementary Table S6) and the Spectacled Whitestart (MYIMEL; Supplementary Table S7).

Supplementary Table S4. Comparisons between the Slate-throated Whitestart (MYIMIN) and the Spectacled Whitestart (MYIMEL) with respect to 10 foraging variables collected on all foraging substrates (CABRTRBU; canopies, branches, trunks and bushes). Results of the Mann-Whitney tests and Wald-Wolfowitz tests. The variable "***Total attack***" is a combination of three variables (pecks, flush-pursuing, flay-catching). Red font indicates significant difference with $p<0.05$. This table corresponds to Fig. 5 of the main text.

	min., lower quartile, **median**, upper quartile, *max.* (*n*=sample size)		Mann-Whitney test			Wald-Wolfowitz test	
	MYIMIN	MYIMEL	*U*	*Z*	*P*	*Z*	*P*
Flights (nr/min)	*0.0*, 3.3, **4.9**, 6.7, *15.0* (39)	*1.0*, 2.8, **3.1**, 4.1, *7.0* (26)	*277.5*	*3.067*	*0.002*	*-0.834*	*0.404*
Hops (nr/min)	*1.8*, 8.4, **10.3**, 15.0, *26.8* (39)	*4.4*, 8.6, **11.4**, 13.9, *27.3* (26)	*491.0*	*-0.208*	*0.836*	*-0.052*	*0.958*
Tail-fanning (nr/min)	*0.0*, 0.0, **0.3**, 2.6, *14.1* (39)	*0.0*, 0.0, **0.0**, 0.9, *7.5* (26)	*440.5*	*0.948*	*0.343*	*0.208*	*0.835*
Tail-fan ratio	*0.0*, 0.0, **0.03**, 0.15, *0.36* (39)	*0.0*, 0.0, **0.0**, 0.08, *0.36* (26)	*448.0*	*0.840*	*0.401*	*0.208*	*0.835*
Pecks (nr/min)	*0.0*, 0.0, **1.5**, 3.7, *7.2* (39)	*0.0*, 0.0, **0.7**, 1.9, *2.6* (26)	*360.5*	*1.980*	*0.048*	*0.469*	*0.639*
Fly-catching (nr/min)	*0.0*, 0.0, **0.0**, 1.5, *3.3* (39)	*0.0*, 0.0, **0.2**, 1.2, *3.2* (26)	*465.0*	*-0.610*	*0.542*	*-2.919*	*0.004*
Flush-pursuit (nr/min)	*0.0*, 0.0, **0.0**, 1.0, *2.8* (39)	*0.0*, 0.0, **0.1**, 0.9, *2.6* (26)	*479.5*	*-0.397*	*0.692*	*1.772*	*0.076*
Tail-fan success	*0.0*, 0.2, **0.5**, 1.0, *1.0* (20)	*0.2*, 0.5, **1.0**, 1.0, *1.0* (12)	*79.5*	*-1.630*	*0.103*	*-1.153*	*0.249*
Hop-pecks success	*0.0*, 0.0, **0.1**, 0.3, *1.6* (39)	*0.0*, 0.0, **0.1**, 0.2, *0.3* (26)	*385.0*	*1.648*	*0.099*	*-1.355*	*0.175*
Total attack	*0.0*, 1.2, **3.2**, 4.7, *9.2* (39)	*0.0*, 1.3, **2.3**, 3.2, *5.0* (26)	*373.0*	*1.790*	*0.074*	*-0.834*	*0.404*

Supplementary Table S5. Comparisons between the Slate-throated Whitestart (MYIMIN) and the Spectacled Whitestart (MYIMEL) with 10 respect to 10 foraging variables collected in canopies only (CA). Results of the Mann-Whitney tests and Wald-Wolfowitz tests. The variable "***Total attack***" is a combination of three variables (pecks, flush-pursuing, flay-catching). Red font indicates significant difference with $p<0.05$.

Variable	*min.*, lower quartile, **median**, upper quartile, *max.* (*n*=sample size)		Mann-Whitney test			Wald-Wolfowitz test	
	MYIMIN	*MYIMEL*	*U*	*Z*	*P*	*Z*	*P*
Flights (nr/min)	*0.0*, 3.4, **5.6**, 6.9, *15.0* (33)	*1.0*, 3.0, **3.3**, 4.5, *7.0* (18)	*157.0*	*2.750*	*0.006*	*-0.402*	*0.688*
Hops (nr/min)	*4.0*, 8.4, **10.3**, 14.7, *23.9* (33)	*4.4*, 8.6, **12.7**, 14.5, *17.7* (18)	*270.0*	*-0.522*	*0.601*	*1.460*	*0.144*
Tail-fanning (nr/min)	*0.0*, 0.0, **0.0**, 1.9, *7.5* (33)	*0.0*, 0.0, **0.2**, 1.0, *7.5* (18)	*285.0*	*0.243*	*0.808*	*0.840*	*0.401*
Tail-fan ratio	*0.0*, 0.0, **0.03**, 0.11, *0.36* (33)	*0.0*, 0.0, **0.03**, 0.09, *0.36* (18)	*293.5*	*0.063*	*0.949*	*0.840*	*0.401*
Pecks (nr/min)	*0.0*, 0.6, **2.1**, 3.8, *7.2* (33)	*0.0*, 0.0, **0.6**, 1.8, *2.6* (18)	*169.0*	*2.540*	*0.011*	*-0.402*	*0.688*
Fly-catching (nr/min)	*0.0*, 0.0, **0.0**, 1.7, *3.3* (33)	*0.0*, 0.0, **0.2**, 1.2, *3.0* (18)	*293.5*	*-0.064*	*0.949*	*-1.022*	*0.307*
Flush-pursuit (nr/min)	*0.0*, 0.0, **0.0**, 0.9, *1.7* (33)	*0.0*, 0.0, **0.6**, 1.0, *2.4* (18)	*259.0*	*-0.081*	*0.423*	*0.840*	*0.401*
Tail-fan success	*0.0*, 0.3, **0.7**, 1.0, *1.0* (16)	*0.2*, 0.8, **1.0**, 1.0, *1.0* (9)	*51.0*	*-1.253*	*0.210*	*0.214*	*0.831*
Hop-pecks success	*0.0*, 0.1, **0.2**, 0.3, *1.2* (33)	*0.0*, 0.0, **0.1**, 0.2, *0.2* (18)	*181.5*	*2.291*	*0.022*	*-1.953*	*0.051*
Total attack	*0.0*, 2.0, **3.6**, 5.3, *9.2* (33)	*0.0*, 1.3, **1.9**, 3.2, *5.0* (18)	*178.5*	*2.328*	*0.020*	*0.840*	*0.401*

Supplementary Table S6. Comparisons of foraging behaviour between foraging substrates for the Slate-throated Whitestart (*M. miniatus*). The variable “***Total attack***” is a combination of three variables (pecks, flush-pursuing, fly-catching). Red font indicates significant difference with p<0.05.

	min., lower quartile, **median**, upper quartile, *max.* (*n*=sample size)		Only one data point collected	*U, Z, P*	(BU) value below lower 5 percentile of CA distribution (Yes/No)	*U, Z, P*
	Canopy only (CA)	Branches + Trunks + Bushes (BRTRBU)	Bushes only (BU)	Mann-Whitney CA <->BRTRBU		Wald-Wolfowitz CA <->BRTRBU
Flights (nr/min)	*0.0*, 3.4, **5.6**, 6.9, *15.0* (33)	*1.3*, 1.8, **4.2**, 4.9, *5.6* (6)	4.6 (1)	*55.0, 1.693, 0.091*	No	*-0.098, 0.922*
Hops (nr/min)	*4.0*, 8.4, **10.3**, 14.7, *23.9* (33)	*1.8*, 9.3, **12.2**, 23.5, *26.8* (6)	1.8 (1)	*81.0, -0.681; 0.505*	No	*-0.098, 0.922*
Tail-fanning (nr/min)	*0.0*, 0.0, **0.0**, 1.9, *7.5* (33)	*0.0*, 0.0, **3.4**, 5.5, *14.1* (6)	0.0 (1)	*59.0, -1,635, 0.102*	Yes	*0.541, 0.588*
Tail-fan ratio	*0.0*, 0.0, **0.03**, 0.11, *0.36* (33)	*0.0*, 0.0, **0.17**, 0.30, *0.34* (6)	0.0 (1)	*60.0, -1.593, 0.111*	Yes	*-0.098, 0.922*
Pecks (nr/min)	*0.0*, 0.6, **2.1**, 3.8, *7.2* (33)	*0.0*, 0.0, **0.3**, 0.7, *0.9* (6)	0.0 (1)	*42.0, 2.218, 0.027*	Yes	*-0.098, 0.922*
Fly-catching (nr/min)	*0.0*, 0.0, **0.0**, 1.7, *3.3* (33)	*0.0*, 0.0, **0.0**, 0.0, *0.9* (6)	0.9 (1)	*68.5, 1.334, 0.182*	Yes	*0.541, 0.588*
Flush-pursuit (nr/min)	*0.0*, 0.0, **0.0**, 0.9, *1.7* (33)	*0.0*, 0.0, **0.0**, 2.4, *2.8* (6)	0.0 (1)	*94.0, -0.196, 0.844*	Yes	*-0.738, 0.461*
Tail-fan success	*0.0*, 0.3, **0.7**, 1.0, *1.0* (16)	*0.0*, 0.0, **0.1**, 0.4, *0.6* (4)	-	*11.5, 1.940, 0.052*	Yes	*1.186, 0.235*
Hop-pecks success	*0.0*, 0.1, **0.2**, 0.3, *1.2* (33)	*0.0*, 0.0, **0.0**, 0.1, *0.1* (6)	0.0 (1)	*34.5, 2.513, 0.012*	Yes	*-0.098, 0.922*
Total attack	*0.0*, 2.0, **3.6**, 5.3, *9.2* (33)	*0.0*, 0.0, **0.9**, 3.0, *3.5* (6)	0.9 (1)	*41.0, 2.240, 0.025*	Yes	*1.180, 0.238*

Supplementary Table S7. Comparisons of foraging behaviour between foraging substrates for the Spectacled Whitestart (*M. melanocephalus*). The variable "***Total attack***" is a combination of three variables (pecks, flush-pursuing, fly-catching).

	min., lower quartile, **median**, upper quartile, *max.* (*n*=sample size)			*U, Z, P*		*Z, P*	
	Canopy only (CA)	Branches + Trunks + Bushes (BRTRBU)	Bushes only (BU)	Mann-Whitney CA <->BRTRBU	Mann-Whitney CA <->BU	Wald-Wolfowitz CA <->BRTRBU	Wald-Wolfowitz CA <->BU
Flights (nr/min)	*1.0*, 3.0, **3.3**, 4.5, *7.0* (18)	*1.9*, 2.4, **2.9**, 3.7, *6.0* (8)	*2.1*, 2.4, **2.9**, 4.6, *6.0* (4)	*56.0, 0.861, 0.397*	*22.5, 1.107, 0.262*	*0.200, 0.841*	*0.726, 0.468*
Hops (nr/min)	*4.4*, 8.6, **12.7**, 14.5, *17.7* (18)	*6.0*, 8.2, **10.7**, 11.7, *27.3* (8)	*6.0*, 7.4, **9.8**, 11.4, *12.0* (4)	*58.5, 0.722; 0.461*	*31.0, 0.383; 0.702*	*0.673, 0.501*	*0.726, 0.468*
Tail-fanning (nr/min)	*0.0*, 0.0, **0.2**, 1.0, *7.5* (18)	*0.0*, 0.0, **0.0**, 0.5, *6.3* (8)	*0.0*, 0.0, **0.0**, 0.0, *0.0* (4)	*58.5, 0,722, 0.432*	*18.0, 1,671, 0.094*	*0.273, 0.785*	*0.726, 0.468*
Tail-fan ratio	*0.0*, 0.0, **0.03**, 0.09, *0.36* (18)	*0.0*, 0.0, **0.0**, 0.05, *0.19* (8)	*0.0*, 0.0, **0.0**, 0.0, *0.0* (4)	*57.5, 0.778, 0.397*	*18.0, 1,671, 0.094*	*1.220, 0.223*	*0.795, 0.426*
Pecks (nr/min)	*0.0*, 0.0, **0.6**, 1.8, *2.6* (18)	*0.0*, 0.1, **1.5**, 2.0, *2.2* (8)	*0.0*, 0.1, **0.8**, 1.8, *2.2* (4)	*59.0, -0.709, 0.478*	*32.5, -0.262, 0.794*	*0.200, 0.841*	*0.726, 0.468*
Fly-catching (nr/min)	*0.0*, 0.0, **0.2**, 1.2, *3.0* (18)	*0.0*, 0.0, **0.1**, 1.7, *3.2* (8)	*0.0*, 0.5, **1.7**, 2.8, *3.2* (4)	*71.0, -0.029, 0.977*	*21.0, -1.278, 0.227*	*0.673, 0.501*	*-0.035, 0.972*
Flush-pursuit (nr/min)	*0.0*, 0.0, **0.6**, 1.0, *2.4* (18)	*0.0*, 0.0, **0.0**, 0.4, *2.6* (8)	*0.0*, 0.0, **0.0**, 0.0, *0.0* (4)	*55.0, 0.980, 0.327*	*16.0, 1.813, 0.098*	*0.273, 0.785*	*0.795, 0.426*
Tail-fan success	*0.2*, 0.8, **1.0**, 1.0, *1.0* (9)	*0.4*, 0.4, **0.5**, 1.0, *1.0* (3)	-	*8.5, 0.930, 0.352*	-	*0.836, 0.403*	-
Hop-pecks success	*0.0*, 0.0, **0.1**, 0.2, *0.2* (18)	*0.0*, 0.0, **0.1**, 0.2, *0.3* (8)	*0.0*, 0.0, **0.1**, 0.2, *3.2* (4)	*60.5, -0.624, 0.532*	*32.0, -0.305, 0.744*	*-0.200, 0.841*	*-0.035, 0.972*
Total attack	*0.0*, 1.3, **1.9**, 3.2, *5.0* (18)	*0.0*, 1.8, **2.6**, 3.3, *4.1* (8)	*1.3*, 1.9, **2.8**, 3.3, *3.4* (4)	*60.0, -0.640, 0.522*	*28.5, -0.597, 0.538*	*1.147, 0.251*	*0.035, 0.972*
